# Diversifying livestock promotes multidiversity and multifunctionality in managed grasslands

**DOI:** 10.1073/pnas.1807354116

**Published:** 2019-03-08

**Authors:** Ling Wang, Manuel Delgado-Baquerizo, Deli Wang, Forest Isbell, Jun Liu, Chao Feng, Jushan Liu, Zhiwei Zhong, Hui Zhu, Xia Yuan, Qing Chang, Chen Liu

**Affiliations:** ^a^Key Laboratory of Vegetation Ecology of Ministry of Education, Institute of Grassland Science, School of Environment, Northeast Normal University, Changchun, 130024 Jilin, China;; ^b^Cooperative Institute for Research in Environmental Sciences, University of Colorado, Boulder, CO 80309;; ^c^Departamento de Biología y Geología, Física y Química Inorgánica, Escuela Superior de Ciencias Experimentales y Tecnología, Universidad Rey Juan Carlos, 28933 Móstoles, Spain;; ^d^Department of Ecology, Evolution, and Behavior, University of Minnesota, Saint Paul, MN 55108

**Keywords:** multiple trophic diversity, ecosystem multifunctionality, grassland grazing management, livestock diversity, mixed grazing

## Abstract

Increasing plant diversity can increase ecosystem functioning, stability, and services in both natural and managed grasslands, but the effects of herbivore diversity, and especially of livestock diversity, remain underexplored. Given that managed grazing is the most extensive land use worldwide, and that land managers can readily change livestock diversity, we experimentally tested how livestock diversification (sheep, cattle, or both) influenced multidiversity (the diversity of plants, insects, soil microbes, and nematodes) and ecosystem multifunctionality (including plant biomass production, plant leaf N and P, above-ground insect abundance, nutrient cycling, soil C stocks, water regulation, and plant–microbe symbiosis) in the world’s largest remaining grassland. We also considered the potential dependence of ecosystem multifunctionality on multidiversity. We found that livestock diversification substantially increased ecosystem multifunctionality by increasing multidiversity. The link between multidiversity and ecosystem multifunctionality was always stronger than the link between single diversity components and functions. Our work provides insights into the importance of multitrophic diversity to maintain multifunctionality in managed ecosystems and suggests that diversifying livestock could promote both multidiversity and ecosystem multifunctionality in an increasingly managed world.

## Significance

The potential importance of herbivore diversity in maintaining ecosystem functioning remains unclear in terrestrial ecosystems. This is a critical knowledge gap because the global human population increasingly relies on grasslands to supply meat and dairy products. As the global human population continues to grow, and as per capita consumption of meat and dairy products continues to increase, livestock grazing will place unprecedented pressures on grasslands worldwide. We show that diversifying livestock could promote grassland biodiversity and ecosystem multifunctionality in an increasingly managed world, and also provide insights into the importance of multitrophic diversity to maintain ecosystem multifunctionality in managed ecosystems. Grassland grazing management by livestock diversification increases nature’s benefits to people, partly by maintaining a diverse array of grassland species.

The strong reduction in biodiversity resulting from habitat loss and climate change has prompted a large body of research to examine the effects of biodiversity loss on ecosystem functioning (1). Most studies have found a strong positive effect of increasing plant diversity on ecosystem functions in terrestrial ecosystems (2–11). However, the role of biodiversity, if any, in driving ecosystem functions in managed ecosystems remains much less explored (but see refs. 12–17). Furthermore, although it is now clear that ecosystem functioning depends even more on herbivore than on plant diversity in aquatic ecosystems (6, 10), the potential importance of herbivore diversity remains unclear in terrestrial ecosystems.

Livestock grazing is the most widespread land use on Earth (18), including in northern China (19), which is part of one of the largest remaining grasslands on Earth (i.e., the Eurasian steppe) where grassland is largely used to support livestock grazing for food production. Livestock grazing can alter both biodiversity and ecosystem functioning (20–22). For example, livestock grazing can directly disturb soils physically (e.g., via soil compaction) and chemically (e.g., altering nutrient cycling via animal dung), thereby affecting plant productivity and ecosystem function. Furthermore, livestock grazing, as an important driver of grassland biodiversity change, not only exerts important and direct effects on plant diversity (23), but also on the diversity of other above-ground and below-ground organisms such as insects (24) and soil animals (25). The increasing human population and per capita demand for the production of meat and animal products (26) has placed tremendous pressures on grassland ecosystems worldwide, including in China. Theory predicts that increasing herbivore diversity could increase the production of herbivores (27) and there is some evidence that mixed grazing can increase livestock production (28, 29). However, the wider impacts of mixed grazing on multitrophic diversity (multidiversity of above-ground and below-ground organisms) and multiple ecosystem functions (multifunctionality) remain completely unexplored. Evaluating the importance of biodiversity in regulating ecosystem function in managed ecosystems is of paramount importance to predict the future dynamics of terrestrial ecosystems in a highly managed world.

Here, we used a 5-y field-manipulated grazing experiment, including livestock grazing by single species (cattle or sheep) and mixed species (sheep and cattle) to evaluate the role of diversifying livestock (single vs. mixed livestock species) in regulating multidiversity, including above-ground (e.g., plants and insects) and below-ground (e.g., microbes and microinvertebrates) organisms, and multifunctionality, including variables related to productivity, nutrient cycling, soil C stocks, water regulation, and plant–microbe symbiosis, and to assess the importance of multidiversity in regulating ecosystem multifunctionality in highly managed ecosystems (*SI Appendix*, Fig. S1).

To obtain a single index reflecting multitrophic diversity (multidiversity), we combined the biodiversity characteristics by averaging the standardized scores [minimum-maximum (min-max) normalization] of species richness across six groups of above-ground and below-ground organisms: plants, herbivorous insects, predatory insects, soil bacteria, fungi, and nematodes (30). We then quantified a multifunctionality index comprising information for 12 above- and below-ground processes, including plant production (plant above- and below-ground biomass), plant nutrient content, and a nutrient source for livestock herbivores (community leaf N and P content), above-ground insect biomass (herbivorous insect and predatory insect abundance), nutrient cycling (in situ measurements of soil N availability, and soil total N and total P), soil C stocks (total organic carbon controlled by bulk density), water regulation (soil moisture), and plant–microbe symbiosis (abundance of soil ectomycorrhizal fungi; see *SI Appendix*, Table S1 for further rationale on the selected functions). These variables constitute a good proxy for productivity, nutrient cycling, and build-up of nutrient pools, which are important determinants of ecosystem functioning in grazing grassland. They also provide information on mycorrhizal colonization (4, 15). Moreover, some of these functions (e.g., plant biomass and leaf nutrient content) are essential for livestock herbivore nutrition (e.g., protein and energy) and are critical for their fitness. The single index of ecosystem multifunctionality (EMF) was quantified by averaging the standardized scores (min-max normalization) of 12 ecosystem functions. We also conducted analyses using a multithreshold multifunctionality approach (7).

## Results and Discussion

**Fig. 1.**
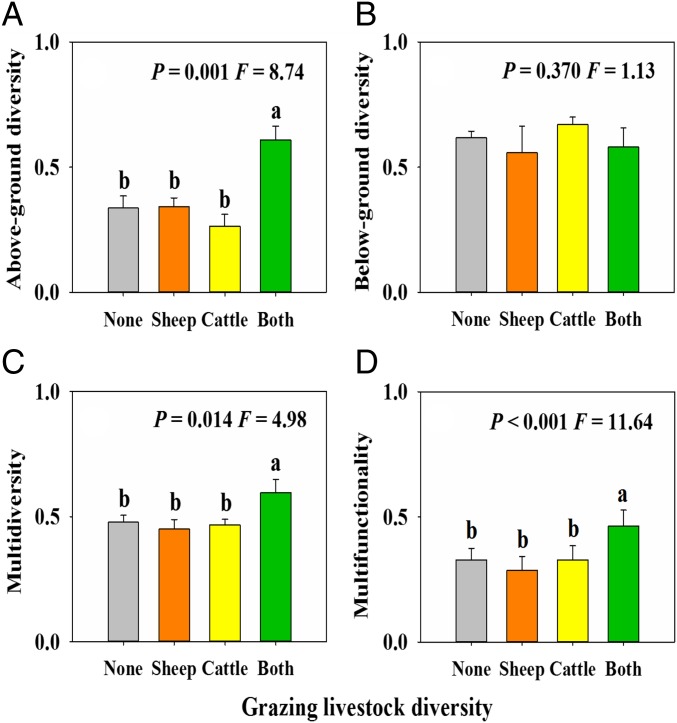
The effects of grazing livestock diversity on above-ground diversity (*A*), below-ground diversity (*B*), multidiversity (*C*), and ecosystem multifunctionality (*D*). Above-ground diversity is calculated as the average value of plant, herbivore, and predator richness after min-max normalization. Below-ground diversity is calculated as the average value of soil bacteria, fungi, and soil nematode richness after min-max normalization. Multidiversity is calculated as the average value of all of the species richness after min-max normalization. Ecosystem multifunctionality is calculated as the average value of herbivorous insect and predatory insect abundance, plant above-ground biomass, below-ground biomass, plant community leaf N and P, soil nitrogen availability, soil total nitrogen and total phosphorus, soil organic C density, soil moisture, and abundance of soil ectomycorrhizal fungi after min-max normalization. Different lowercase letters within panels indicate significant (*P* < 0.1) differences between treatment means, after using Tukey’s method to correct for multiple comparisons. Error bars represent ±1SE.

Our results provide experimental evidence that diversifying livestock has the potential to increase multidiversity and multifunctionality in managed ecosystems. Here, we show that increasing from one to two species of livestock significantly and substantially increases above-ground diversity, multidiversity, multifunctionality (Fig. 1), and also the weighted EMF (*SI Appendix*, Fig. S12). Cattle and sheep exhibit distinctive feeding modes and preferences (31), and therefore could have a synergistic and complementary effect on vegetation structure (32). Furthermore, diversifying livestock may provide a wider variety of niches for insects and soil organisms compared with single livestock, for example, by increasing the types of animal dungs and plant litter. This result suggests that mixing livestock species, at a given animal density, could be potentially used as a tool for managing grasslands to conserve biodiversity, to regulate multiple ecosystem services, and to promote and sustain human well-being. As such, we argue that slight changes in grazing management (e.g., increasing diversity of herbivores, under a similar grazing intensity level) could favor biodiversity and multifunctionality in an increasingly managed world. Here, we focused on two of the most abundant livestock herbivores on Earth, and in China: cattle and sheep (33). Future studies will be needed to evaluate the potential contribution of further increasing livestock diversity beyond these two species or of mixing other livestock species (e.g., goats and horses) and varieties (e.g., different breeds of cattle or sheep) for increasing multidiversity and multifunctionality in China and elsewhere. Our results suggest that it may be possible to indirectly manage grassland biodiversity through livestock management, which would likely be less difficult and costly than directly managing the diversity of plants, insects, and microbes. Such livestock management could help to conserve multiple ecosystem functions and types of organisms, which threaten the sustainability of terrestrial ecosystems worldwide, especially in developing countries. Our findings suggest that diversifying livestock grazing might not only directly provide livestock products and increase the amount of biomass and the nutrient content of forage for livestock, but also, more importantly, help improve the biodiversity and ecosystem functioning of these economically and ecologically important regions.

**Fig. 2.**
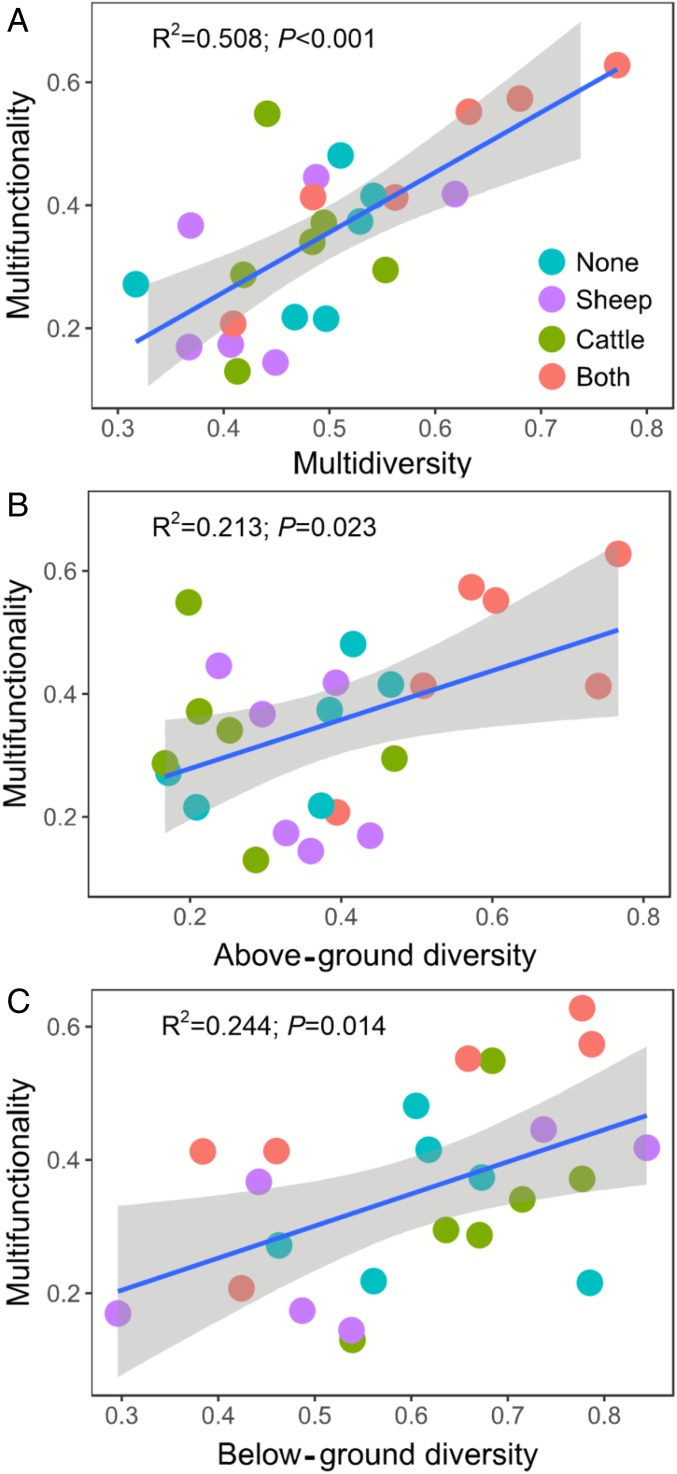
Relationships between ecosystem multifunctionality and multidiversity (*A*), above-ground diversity (*B*), and below-ground diversity (*C*). Ecosystem multifunctionality is calculated as the average value of 12 ecosystem functions (see *SI Appendix*, Table S1 for list) after min-max normalization. The blue fitted lines are from ordinary least squares regression.

Critically, we also found a strong positive relationship between multidiversity and multifunctionality in this managed ecosystem (Fig. 2*A*). Similar positive and significant relationships between multidiversity and the number of functions over different thresholds were observed here for the 25%, 50%, 75%, and 90% thresholds when we used a multithreshold multifunctionality approach (*SI Appendix*, Fig. S2). This positive relationship was also observed when we recalculated our multifunctionality index to down-weight highly correlated functions as described in Manning et al. (34) (*SI Appendix*, Figs. S9–S11), suggesting that our results are robust to the choice of multifunctionality index. In addition, the association between multidiversity and multifunctionality was stronger than the correlations between above- or below-ground diversity and multifunctionality (Fig. 2 *B* and *C*). The significant effects of multidiversity and above- or below-ground diversity in driving multifunctionality were also maintained after accounting for soil environmental conditions (soil pH, electrical conductivity, and bulk density), and grazing management types (livestock diversity) using random forest modeling (*Materials and Methods*) (*SI Appendix*, Fig. S3).

**Fig. 3.**
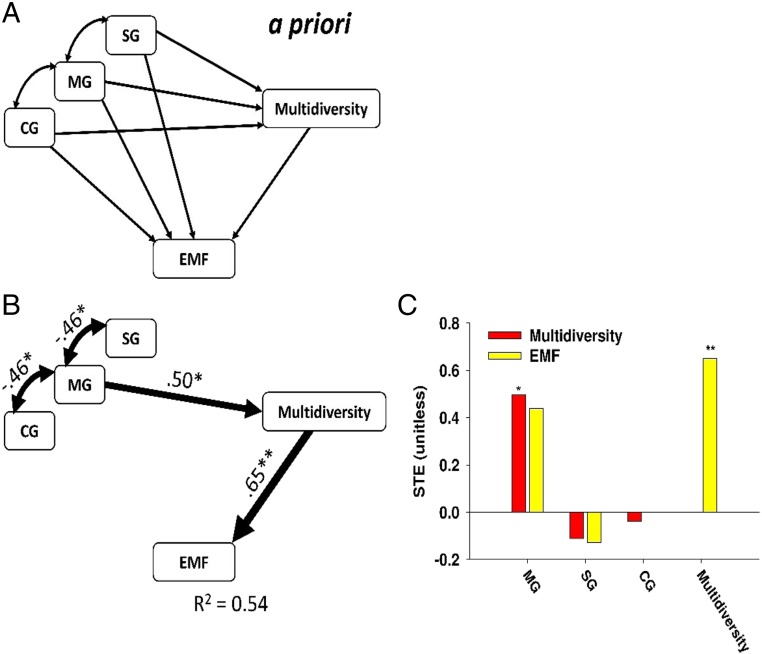
Direct and indirect effects of livestock diversity on EMF. (*A*) Hypothesis model, (*B*) structural equation model describing the effects of grazing livestock diversity and biodiversity on ecosystem multifunctionality, and (*C*) standardized total effects (direct plus indirect effects) derived from the structural equation model depicted. Numbers adjacent to arrows are indicative of the effect size of the relationship. R^2^ denotes the proportion of variance explained. Significance levels of each predictor are *P < 0.05, **P < 0.01. STE, standardized total effects. There was a nonsignificant deviation of the data from the model [χ^2^ = 2.71, df = 1; *P* = 0.10; root mean square error of approximation (RMSEA) *P* = 0.11].

**Fig. 4.**
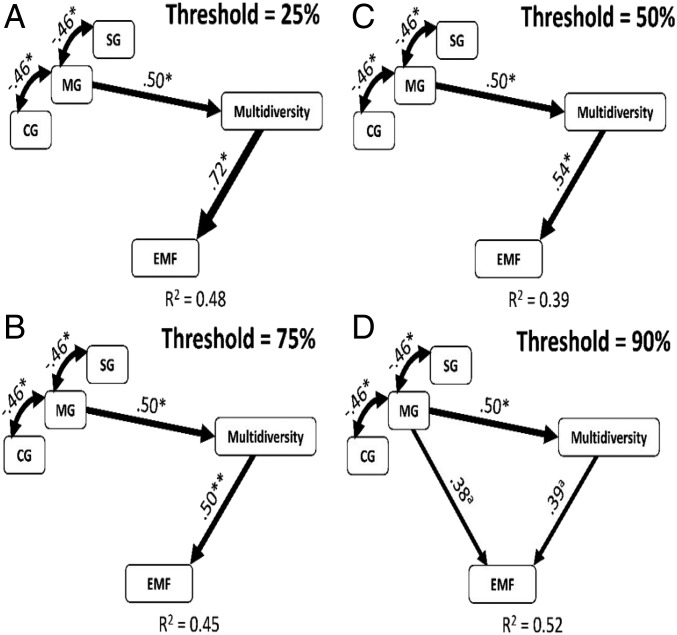
Structural equation model describing the effects of grazing livestock diversity and biodiversity on the number of functions beyond different thresholds of 25% (*A*), 50% (*B*), 75% (*C*), and 90% (*D*), calculated following the multithreshold approach. Numbers adjacent to arrows are indicative of the effect size of the relationship. R^2^ denotes the proportion of variance explained. Significance levels of each predictor are ^a^*P* < 0.10, *P < 0.05, **P < 0.01. STE, standardized total effects (sum of direct and indirect effects). In all cases, there was a nonsignificant deviation of the data from the model (χ^2^ = 2.71, df = 1; *P* = 0.10; RMSEA *P* = 0.11).

We then conducted structural equation modeling (SEM) to evaluate the possible existence of direct effects of grazing livestock diversity on multifunctionality, as well as indirect effects that are mediated by changes in multidiversity. We found that mixed livestock indirectly increased multifunctionality by promoting multidiversity (Fig. 3). Similar results were found when using a multithreshold multifunctionality approach (Fig. 4). These findings indicate that livestock diversity positively, but indirectly drives multifunctionality by increasing multidiversity. Specifically, livestock diversity drives multifunctionality mainly by mediating above-ground diversity (*SI Appendix*, Fig. S4), because below-ground diversity was not significantly affected by livestock diversity (Fig. 1). Further, in considering each component of above-ground biodiversity, we found that insect richness showed strong positive effects on EMF (*SI Appendix*, Table S2). The diversity of insects is a major controller of multifunctionality by regulating key ecosystem processes such as litter and organic matter decomposition, which in turn, provides substrate to other important soil organisms involved in nutrient cycling and climate regulation, such as bacteria and fungi (35, 36). We therefore suggest that diversifying livestock could be a plausible in situ strategy to maintain multifunctionality in managed ecosystems by promoting multidiversity, especially the diversity of insects (*SI Appendix*, Fig. S6 and Table S2).

Multidiversity and multifunctionality were more positively and significantly related to each other when multiple groups of above-ground organisms (plants and insects) and below-ground (microbes and nematodes) were considered simultaneously. That is, the relationship between biodiversity and ecosystem function was more positive and significant as more functions and more trophic levels were considered (*SI Appendix*, Table S2). In considering each component of ecosystem, below-ground soil bacterial richness showed the strongest positive effects on most individual functions measured, as well as EMF (*R* = 0.647, *SI Appendix*, Table S2). The empirical evidence for a strong link between soil biodiversity and multiple ecosystem functions is growing (5, 7, 37). However, the effect of multidiversity on multifunctionality was not driven by this single group of organisms because the relationship was maintained even after excluding soil bacterial diversity from our analyses (*R* = 0.599, *SI Appendix*, Table S2). These results are consistent with those of some previous studies that also found positive relationships between multifunctionality and multidiversity (15). Uniquely, our results also experimentally demonstrate how livestock diversification can be used to enhance multifunctionality by increasing multidiversity.

Together, our work provides experimental evidence that diversifying livestock can increase ecosystem multifunctionality by promoting multidiversity, especially the diversity of above-ground insects and plants, which fuel the ecosystem via their debris inputs. Our work suggests that species loss across many trophic groups could have a negative impact on the functioning of these important ecosystems for human well-being. Also, our study suggests that diverse livestock grazing management practices could help promote biodiversity, thereby promoting multiple ecosystem functions in highly managed grasslands.

## Materials and Methods

### Study Site.

Our study was conducted in a semiarid meadow steppe at the Grassland Ecological Research Station of Northeast Normal University, Jilin Province, People’s Republic of China (44°40′–44°44′N, 123°44′–123°47′E). This area is part of one of the largest remaining grasslands on Earth. Mean annual temperature and precipitation (2004–2013) are 6.1 °C and 393.0 mm, respectively (Changling County Climate Station, Jilin Province). Vegetation is dominated by the grass *Leymus chinensis* Tzvel, a common perennial species in the eastern Eurasian steppes. Other common species include but are not limited to the graminoids *Phragmites australis* Trin., *Calamagrostis epigejos* Roth., *Chloris virgata* Swartz, *Carex duriuscula* C. A. M.; the forbs *Kalimeris integrifolia* Turcz., *Potentilla flagellaris* Willd. Ex Schlecht., *Artemisia scoparia* Waldstem et Kitailael; and two legumes, *Lespedeza davurica* Schindl and *Medicago ruthenica* C. W. Chang. All these plant species are edible for cattle and sheep, and the dominant grass—*L. chinensis*—is relative low quality due to high fiber, while some forbs are relative high quality due to high protein content.

### Experimental Design.

A long-term grazing experiment was initiated in 2008 with a completely randomized block design. Six sites (blocks) were selected. Each site was divided into four plots, to which grazing treatments were randomized: no grazing (NG), sheep grazing (SG), cattle grazing (CG), and mixed grazing by sheep and cattle (MG). The same livestock biomass per unit area was applied in all of the grazing treatments to control grazing intensity while testing the effects of livestock diversity on biodiversity and ecosystem function. Sheep grazing plots were subject to grazing by 16 2-y-old northeast fine-wool sheep (body weight 32.0 ± 1.8 kg, mean ± SE) and cattle grazing plots were subject to grazing by 4 adult Simmental beef cattle (body weight 300.0 ± 7.5 kg, mean ± SE) described in Liu et al. (31). The number of cattle and sheep in each group were selected to provide comparable grazing intensity across livestock treatments. Based on their daily intake during pretrials (about 1.5 ± 0.3 kg and 6.0 ± 0.8 kg of forage eaten by sheep and cattle, respectively), we assume that 4 adult sheep are equivalent to 1 beef cow. The mixed cattle and sheep grazing plots included 16 2-y-old northeast fine-wool sheep and 4 adult Simmental beef cattle. Plots for the NG, SG, and CG were 25 m × 25 m in size, while plots for MG were 25 m × 50 m. Mixed grazing plots were twice as large as other plots to ensure equal grazing intensity between single and mixed grazing treatments, while also maintaining herd size for each livestock species. We used a 25 m × 50 m plot including 16 sheep and 4 cattle for the mixed grazing treatment (i.e., instead of 8 sheep and 2 cattle in 25 m × 25 m plots) as herd size is known to strongly influence livestock behavior (38). For example, it has been shown that in small groups, animals interrupt their foraging to scan the environment more frequently, which thus reduces time spent foraging, i.e., animal foraging efficiency (38). Overall, grazing was maintained at a moderate intensity (i.e., 6.67 sheep units per ha^−1^) in each livestock treatment. We used the same set of cattle and sheep in all of the grazing treatments, i.e., rotational grazing treatment to avoid effects of using different individual animals in different treatments on diversity and functions. Each year, grazing occurred from June to September. To ensure that these differences in plot size (between MG and other treatments) had no direct effect on the mean or variance of samples collected, all sampling was conducted at the same spatial scales and within the same total area in each of the four livestock treatments.

### Biodiversity.

#### Above ground: Vegetation investigation.

We conducted vegetation sampling in mid-August 2012. We established three 25-m parallel transects at 6.25-m intervals within each plot. Then, we located 50 cm × 50 cm quadrats along each transect at 5-m intervals. We used these 15 quadrates to measure plant diversity (richness) in each plot.

#### Above ground: Insect sampling and identification.

Insect sampling was carried out four times from early July to late September in 2012, using two complementary sampling methods: sweep netting and suctions (39, 40). We used sweep netting to sample insects by using a light muslin net along two 2-m wide and 25-m long parallel transects within each plot (41, 42). Each sampling was composed of 15 sweeps in each transect, and two samplings were carried out in each plot to ensure that those samples were representative on each sampling date. In addition, we used a “d-Vac” suction sampler (John W. Hock Company) equipped with a circular nozzle of 15.6-cm diameter to sample insects. A total of 16 sampling points were placed at the intersections of a grid formed by squares of 5 m-length sides that was superimposed upon a map of each experimental plot. The precise coordinates of each sampling point (intersection) was located in each plot using a geographical positioning system (GPS) handset. The area of each sampling point was 1 m × 1 m. Each sample consisted of three pooled subsamples taken for each sampling point. Each subsample was taken with a 50-s suction time. Insect specimens were collected under favorable monitoring conditions (sunny days with minimal cloud cover and calm or no wind), from 9:00 AM to 15:00 PM. All plots were visited on the same day and in random order on each sampling date. The contents of the sweep net were preserved in bottles containing ethyl acetate. All individuals were identified to species level (morphospecies), and specimens that could not be identified to species were separated into recognizable taxonomic units. Nymphs, larvae, and other immature insects were not considered due to problems of species identification (3.72% of samples). All herbivorous and predatory insect specimens were recognized based on mouth parts, knowledge of their natural histories, and consultation with the literature. Species richness of herbivores and predators for each experimental plot included all species sampled throughout the sampling period in a given experimental year.

#### Below ground: Soil bacteria and fungi.

Nine of the 15 quadrats (explained above for plant richness) were randomly selected in each plot for soil sampling in mid-August 2012. A composite sample (that is, from five soil samples; top 15 cm) was taken per quadrat. Each sample was separated into three portions. The first portion was air dried for edaphic properties analysis (i.e., soil organic C, total N, total P, and soil moisture). The second portion was archived at −80 °C for microbial diversity and composition analysis. The third portion was directly used for soil nematode extraction.

Genomic DNA was extracted from 500 mg of soil for each sample using the E.Z.N.A. Soil DNA Kit (Omega Bio-Tek, Inc.) according to the manufacturer’s instructions (43). Subsequently, all extracted DNA samples were stored at −20 °C before PCR amplification.

Targeted amplification of bacterial 16S rRNA and fungal ITS sequences were performed to characterize soil microbial community diversity. The V1–V3 region of the 16S rRNA genes was amplified with 27F (44) and 533R primers containing *A* and *B* sequencing adapters (454 Life Science). The forward primer (*B*-27F) was 5′-*CCTATCCCCTGTGTGCCTTGGCAGTCGACT*AGAGTTTGATCCTGGCTCAG-3′, with the sequence of the *B* adapter in italics. The reverse primer (*A*-533R) was 5′-*CCATCTCATCCCTGCGTGTCTCCGACGACT*NNNNNNNNNNNNTTACCGCGGCTGCTGGCAC-3′, with the sequence of the *A* adapter in italics and Ns denoting a unique 12-bp error-correcting Golay barcode used to tag each PCR product. For each sample, PCR reactions for bacteria were carried out in triplicate 20-μL reactions with 0.4 μL of each primer at 5 μmol⋅L^−1^, 10 ng template DNA, 2 μL dNTPs at 2.5 mmol⋅L^−1^, 0.4 μL FastPfu Polymerase (TransGen AP221-02: TransStart FastPfu DNA Polymerase; TransGen Biotech), 4 μL 5× FastPfu buffer, and certified DNA-free PCR water, according to the following procedures: 95 °C for 2 min; 25 cycles of 95 °C for 30 s, 55 °C for 30 s, 72 °C for 30 s, and 72 °C for 5 min. Primers ITS1 and ITS4 amplified the ITS region (45), also containing *A* and *B* sequencing adapters (454 Life Science). The forward primer (*B*-ITS4) was 5′- *CCTATCCCCTGTGTGCCTTGGCAGTCGACT*TCCTCCGCTTATTGATATGC-3′. The reverse primer (*A*-ITS1) was 5′- *CCATCTCATCCCTGCGTGTCTCCGACGACT*NNNNNNNNNNNNTCCGTAGGTGAACCTGCGG-3′. PCR reactions for each sample were carried out in triplicate 20-μL reactions with 0.8 μL of each primer at 5 μmol⋅L^−1^, 10 ng template DNA, 2 μL dNTPs at 2.5 mmol⋅L^−1^, 0.4 μL FastPfu Polymerase (TransGen AP221-02; TransStart FastPfu DNA Polymerase; TransGen Biotech), 4 μL 5× FastPfu buffer, and certified DNA-free PCR water, according to the following procedures: 95 °C for 2 min, 32 cycles of 95 °C for 30 s, 53 °C for 30 s and 72 °C for 30 s, and 72 °C for 5 min. Bacterial and fungal PCR amplifications were all performed on the ABI GeneAmp 9700 PCR system (Applied Biosystems). Then, replicated amplicons were pooled and visualized on 2% agarose gels using SYBR Safe DNA gel stain in 0.5× TBE. Subsequently, amplicons were cleaned using the AxyPrep DNA Gel Extraction Kit (Axygen Biosciences), quantified by PicoGreen dsDNA Quantitation Reagent and QuantiFluor-ST Fluorometer (Promega Corp.), and combined with equimolar ratios into a single tube. The barcoded pyrosequencing for bacteria and fungi was performed on a 454 GS FLX System platform (Roche 454 Life Science) at the Shanghai Majorbio Bio-Pharm Technology Co., Ltd., Shanghai, China.

The pyrosequencing reads were analyzed using Quantitative Insights Into Microbial Ecology (QIIME, qiime.org/), and the details of the analysis pipeline used followed the procedure described in Hamady et al. (46). In our study, sequences more than 200 bp in length with an average quality score >25 and without ambiguous base calls were included in the subsequent analyses. The 12-bp barcode was examined to assign sequences to soil samples. Usearch (version 7.1, qiime.org/) was used to check for chimeras and to cluster the trimmed and unique sequences into operational taxonomic units (OTUs) at the 97% similarity level (47, 48). Phylotype richness (number of unique OTUs) across all samples of the microbial community diversity was calculated. For calculations for the diversity metrics, samples were rarified to 3,000 sequences for bacteria and 2,438 sequences for fungi per soil sample.

#### Below ground: Soil nematode extraction and identification.

Nematodes were extracted for 48 h from 50 g fresh soil using the Baermann funnel method (49). After extraction, nematodes were heat killed with 80 °C hot water to achieve elongation of the nematodes and fixed in 4% formaldehyde. A minimum of 100 individuals (or all if below) were identified to genus level using 100× magnification (50).

### Ecosystem Functions.

#### Plant biomass and community leaf N and P.

Five of the 15 quadrats (explained above) were randomly selected in each plot for quantifying plant biomass. To do so, we harvested all of the above-ground biomass (>2.5 cm above soil surface) in these five quadrats. The live plant samples were separated into leaf and shoot, and oven dried at 65 °C for 48 h and weighed. Then, we ground the above-ground leaf materials to a fine powder on a ball mill and analyzed for plant nitrogen and phosphorus. Leaf N content was analyzed using the Kjeldahl method (A 2300 Kjeltec Analyzer Unit; Foss Tecator), and leaf P content was analyzed using fully automated high technology discrete analyzer (Smartchem 450; AMS) after H_2_SO_4_-H_2_O_2_ digestion. We then collected below-ground root biomass to a depth of 30 cm using soil cores (diameter 7 cm) in each of these five quadrats as well. Roots were collected by rinsing the samples using sieves (mesh size 0.25 mm) on the same day, and then oven dried at 65 °C for 48 h and weighed.

#### Insect abundance.

We recorded the accumulative abundance of insect herbivores and predators throughout sampling periods (explained above for insect sampling and identification).

#### Soil variables.

Soil samples for the measurement of soil organic C, total N, total P, and soil moisture were collected in mid-August 2012 (explained above). Soil organic C was determined with the K_2_Cr_2_O_7_ titration method after digestion (51). Soil N was determined by Kjeltec 2300 Analyzer Unit (FOSS) after wet digestion with H_2_SO_4_ plus catalyzer CuSO_4_ and K_2_SO_4_ (52). Soil P was measured by the HClO_4_-H_2_SO_4_ digestion method (53). Soil moisture was determined gravimetrically using 10 g of fresh soil samples dried at 105 °C for 24 h to a constant weight. Soil N availability was determined in July–August 2013 using ion-exchange resin membranes (Ionics), which were made from anion and cation sheets that were cut into 2.5 cm × 10 cm strips described in Liu et al. (54). Membrane strips were pretreated using 0.5M HCl and 0.5 M NaHCO_3_ to remove existing nutrient ions. We inserted one anion and one cation strip in each sampling quadrat to absorb nitrate ions (NO_3_^−^) and ammonium ions (NH_4_^+^), respectively. After 15 d, we collected membranes and then immediately rinsed each with deionized water to remove soil. Membranes were placed in polyethylene bags with ∼20 mL deionized water, then transported to the laboratory in an ice-filled cooler and stored at 4 °C until analysis. To extract NH_4_^+^ and NO_3_^−^ from the membranes, each pair of membranes was placed in a 250-mL conical flask with 70 mL 2N KCl and shaken at 40 rpm for 1 h using a reciprocal shaker before being filtered through a 1-μm Whatman glass filter. NH_4_^+^ and NO_3_^−^were analyzed with an Alliance Flow Analyzer (Futura). Soil NH_4_^+^ and NO_3_^−^ were calculated by the formula: [(conc in µg N per mL) × 70 mL KCl)]/(50 cm^2^ area of the strip × days in the ground). Soil N availability was determined as the sum of ammonium and nitrate extracted from the membrane pair. Information on ectomycorrhizal fungi was obtained from the online application FUNGuild described in Nguyen et al. (55). The relative abundance of ectomycorrhizal fungi was calculated as the sum of the relative abundance of all taxa (OTUs) sharing that particular functional group.

#### Assessing ecosystem multifunctionality and biodiversity.

We used 12 variables reflecting ecosystem multifunctionality including above-ground herbivorous insect abundance and predatory insect abundance, above-ground plant biomass, plant community leaf N and P, and below-ground root biomass, soil total N and P, and soil N availability, soil organic C density, soil moisture, and abundance of soil ectomycorrhizal fungi (*SI Appendix*, Fig. S1 and Table S1). We then calculated the average multifunctionality index. This index is widely used in the multifunctionality literature (6, 7, 56–58). Moreover, we also calculated the number of functions beyond a given threshold (25%, 50%, 75%, and 90%) using the multithreshold approach described in Byrnes et al. (57), as explained in Delgado-Baquerizo et al. (7). Before analyses, all individual ecosystem function (EF) variables were standardized by transformation as follows: EF = [rawEF − min(rawEF)]/[max(rawEF) − min(rawEF)], with EF indicating the final (transformed) ecosystem function value and raw EF indicating raw (untransformed) ecosystem function values. This way each transformed EF variable had a minimum value of zero and a maximum of 1. Theses standardized ecosystem functions were then averaged to obtain a multifunctionality index (4). Moreover, we calculated the weighted EMF to down-weight highly correlated functions as described in Manning et al. (34). We combined the biodiversity characteristics (plant richness, herbivorous and predatory insect richness, soil bacterial richness and fungal richness, and nematode richness) in the same manner to obtain a single index reflecting a synthetic whole-ecosystem biodiversity measure multidiversity, which integrated information on a wide diversity of groups of organisms. Moreover, we calculated above-ground multidiversity and below-ground multidiversity, respectively, but also repeated some of our analyses for single functions (*SI Appendix*, Table S2).

#### Statistical analyses.

The effects of grazing livestock diversity on above- and below-ground diversity, multidiversity, ecosystem functions, and EMF were analyzed with a two-way ANOVA, with livestock diversity as the main factor, and block as the random factor. Tukey’s multiple comparisons were used as a post hoc analysis to test for significant differences among all treatments. The analyses were carried out in SPSS software version 17.0.

### Random Forest.

Using the rfPermute R package, we conducted a classification random forest analysis to identify which factors were the main predictors of multifunctionality among the following variables: grazing management (livestock diversity), soil bulk density, soil pH, electrical conductivity, and multidiversity (or above- or below-ground diversity) (59). This random forest analysis has been used to identify the major predictors of multifunctionality in Delgado-Baquerizo et al. (7).

### Structural Equation Modeling.

We used SEM to evaluate the direct and indirect effects of grazing livestock diversity on multifunctionality and number of functions beyond a given threshold (25%, 50%, 75%, and 90%) using the multithreshold approach. In all cases the livestock diversity were categorical variables with two levels: 1 (a particular management type: cattle, sheep, and cattle + sheep) and 0 (remaining management types + control). The goodness of fit of SEM models was checked using the following: the χ^2^ test and the Bollen–Stine bootstrap test as done in Delgado-Baquerizo et al. (7). We also repeated our analyses using as a response variable the residuals from an ANOVA using averaging EMF as our response variable and block as a predictor. The aim for this analysis is to ensure that block design is not influencing our results on the effects of grazing livestock diversity and biodiversity on EMF (*SI Appendix*, Fig. S5). SEM models were conducted with the software AMOS 20 (IBM SPSS, Inc.).

## Supplementary Material

Supplementary File
