# Supplementary material for: Diversifying livestock promotes multidiversity and multifunctionality in managed grasslands
Source: Proc Natl Acad Sci U S A. 2019 Mar 8;116(13):6187–92. doi: 10.1073/pnas.1807354116 (PMC6442565; doi:10.1073/pnas.1807354116)
Supplement: Supplementary File [file pnas.1807354116.sapp.pdf]

PNAS
www.pnas.org

Supplementary Information for

Diversifying livestock promotes multidiversity and multifunctionality in managed grasslands

Ling Wang[a], Manuel Delgado-Baquerizo[b,c], Deli Wang[a,1], Forest Isbell[d], Jun Liu[a], Chao Feng[a], Jushan Liu[a], Zhiwei Zhong[a], Hui Zhu[a], Xia Yuan[a], Qing Chang[a], Chen Liu[a]

Deli Wang
Email: wangd@nenu.edu.cn

This PDF file includes:

Figs. S1 to S12
Tables S1 to S2
References for SI reference citations

www.pnas.org/cgi/doi/10.1073/pnas.1807354116

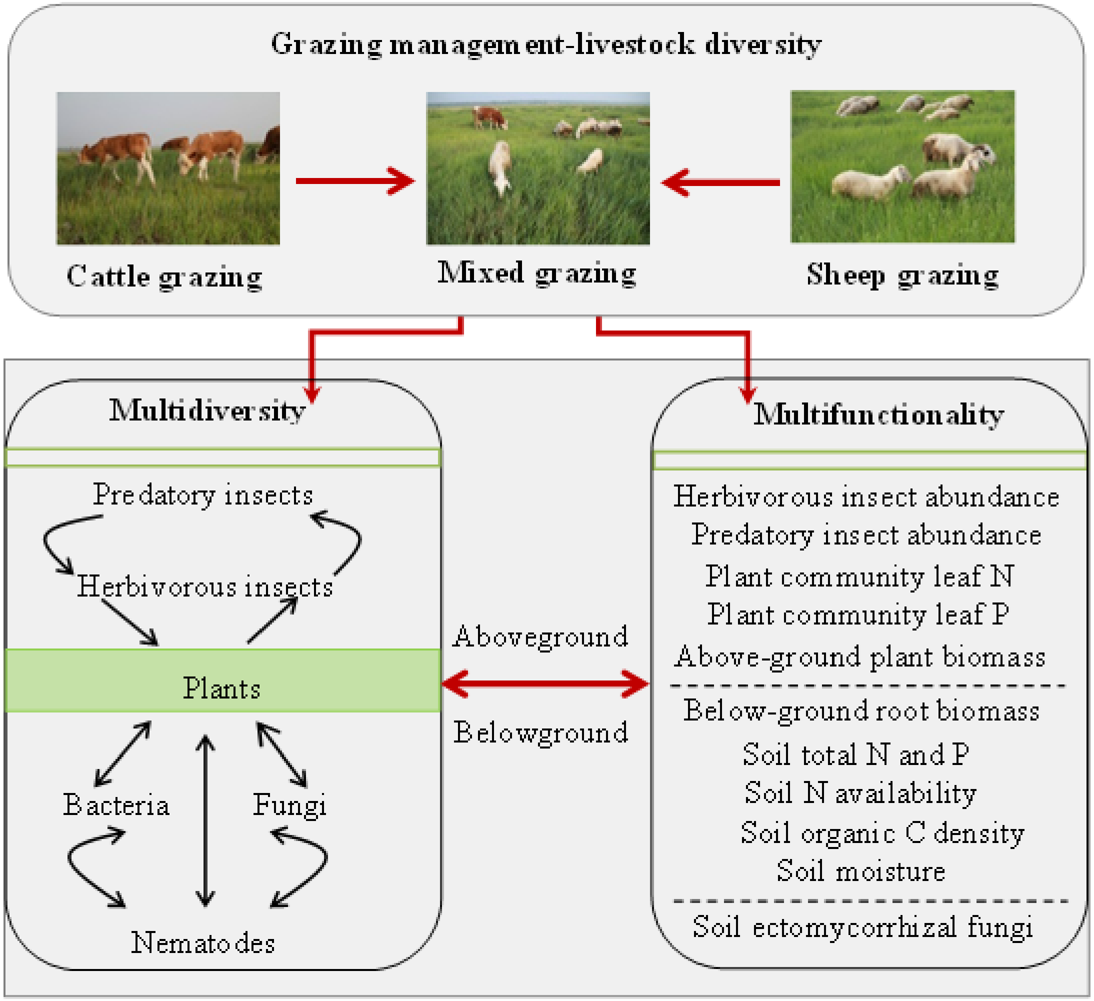

Fig. S1. A framework for testing effects of grazing livestock diversity on multitrophic diversity and ecosystem multifunctionality. See Table S1 for theoretical information on all 12 functions and their importance.

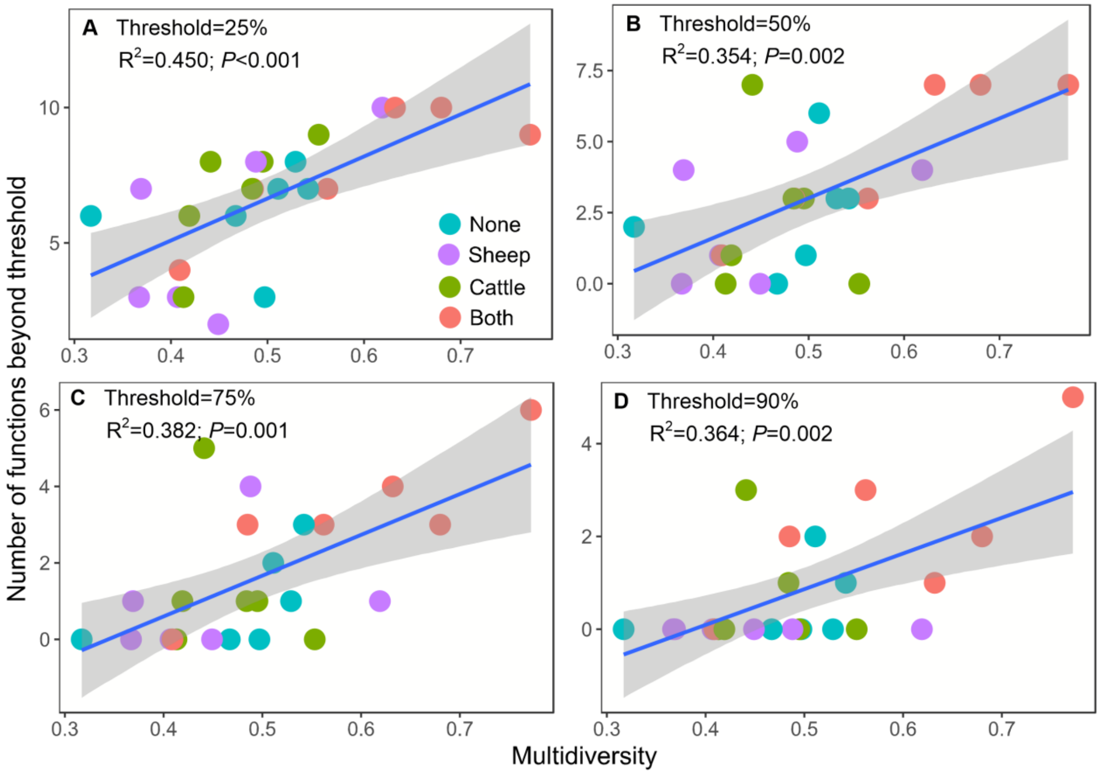

Fig. S2. Relationships between multidiversity and the number of functions beyond different thresholds of 25% (*A*), 50% (*B*), 75% (*C*) and 90% (*D*) calculated following the multi-threshold approach. The blue fitted lines are from OLS regression.

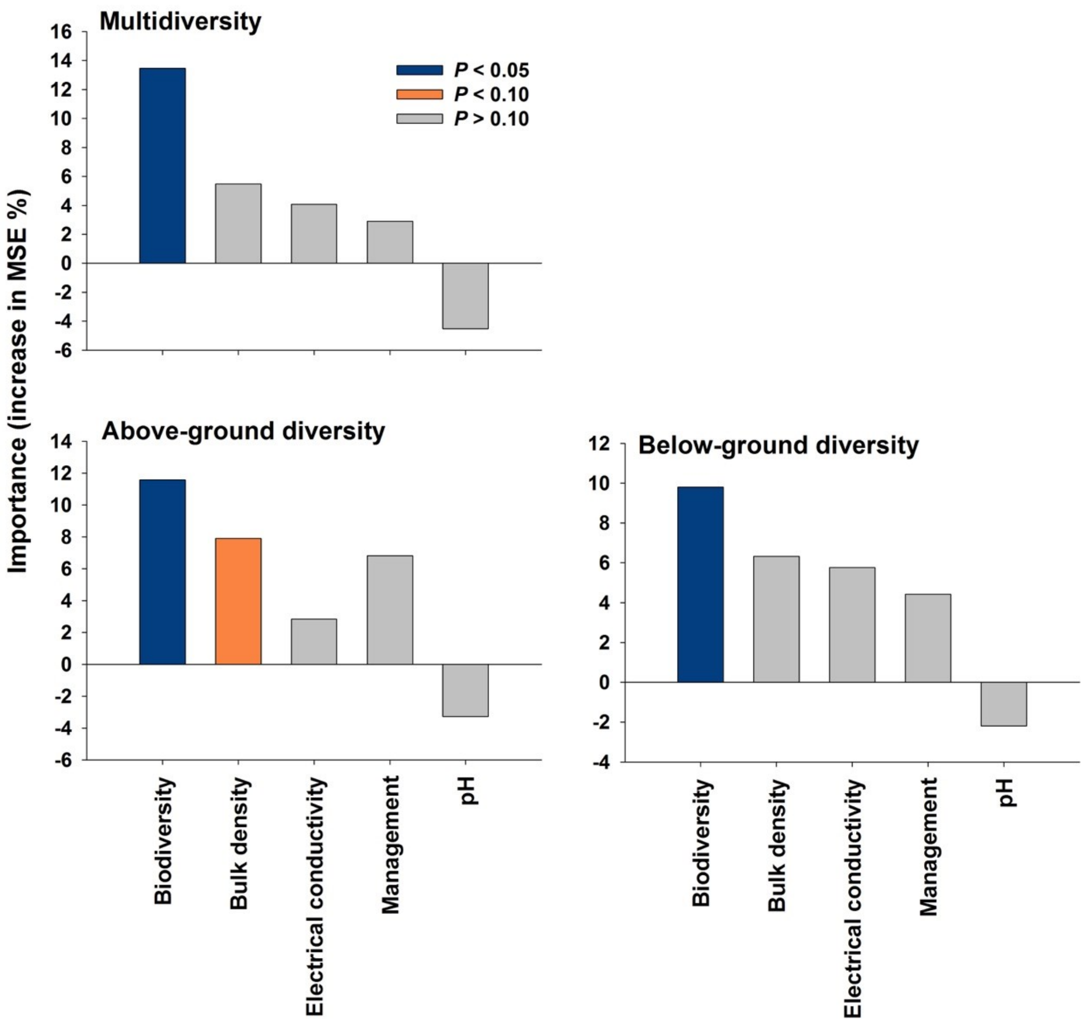

Fig. S3. Main predictors of ecosystem multifunctionality. The figure shows the Random Forest mean predictor importance (% of increase of MSE) of grazing management (i.e. grazing livestock diversity), soil environmental drivers and biodiversity on ecosystem multifunctionality for the above-ground diversity, below-ground diversity and multidiversity data sets. Significant levels of each predictor are shown.

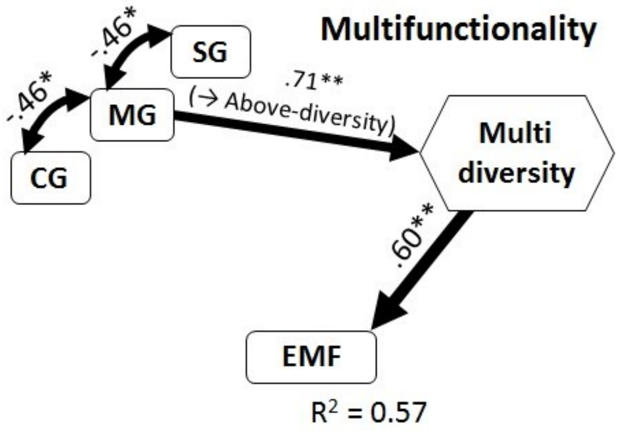

Fig. S4. Structural equation model describing the effects of grazing livestock diversity on ecosystem multifunctionality (EMF) by above- or below-ground diversity. The hexagon represents composite variables including above- and below-ground diversity (multidiversity).

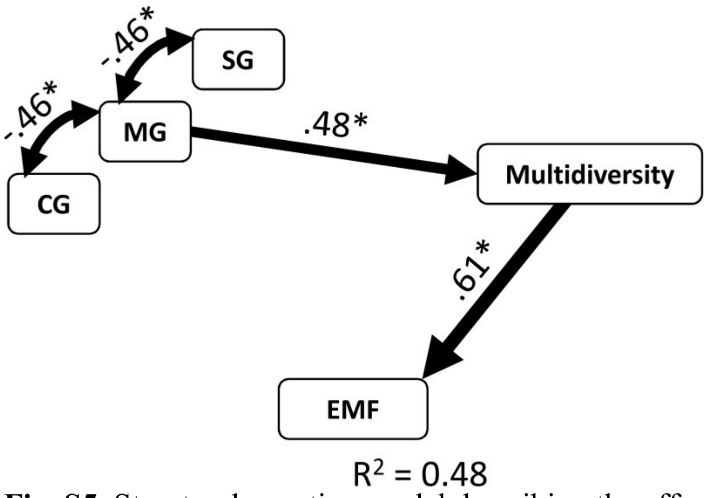

Fig. S5. Structural equation model describing the effects of grazing livestock diversity and biodiversity on ecosystem multifunctionality including the residuals of an ANOVA including block as a predictor and EMF as the response variable.

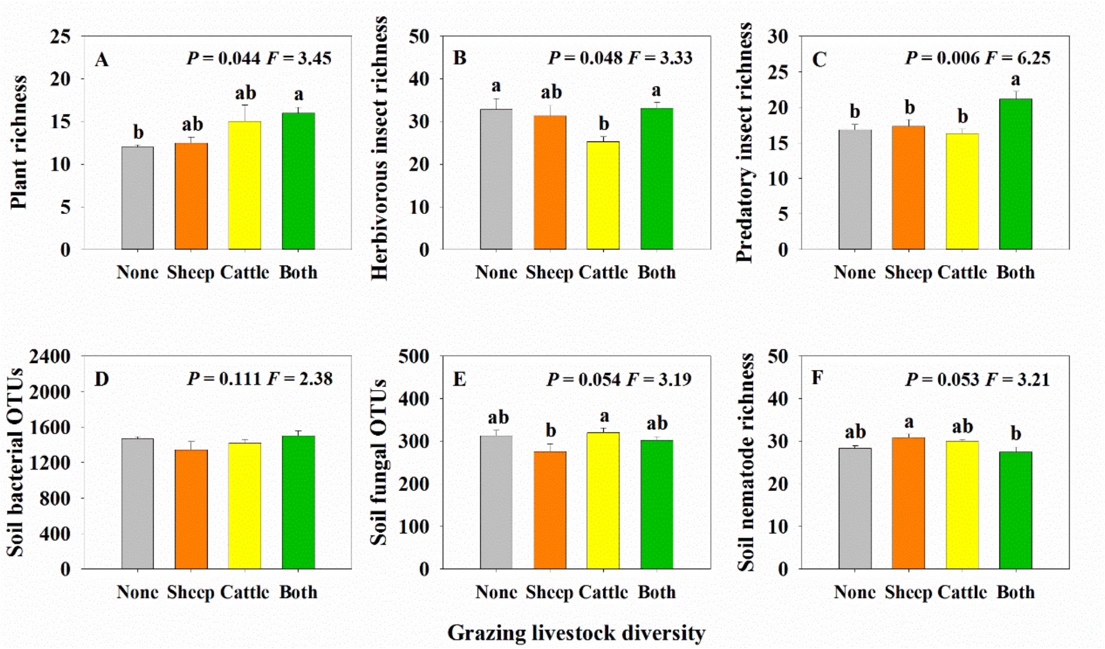

Fig. S6. The effects of grazing livestock diversity on plant richness (*A*), herbivorous insect richness (*B*), predatory insect richness (*C*), soil bacterial OTUs (*D*), soil fungal OTUs (*E*), soil nematode richness (*F*). Different lowercase letters within panels indicate significant ($P < 0.1$) differences between treatment means, after using Tukey's method to correct for multiple comparisons. Error bars represent ±1 SE.

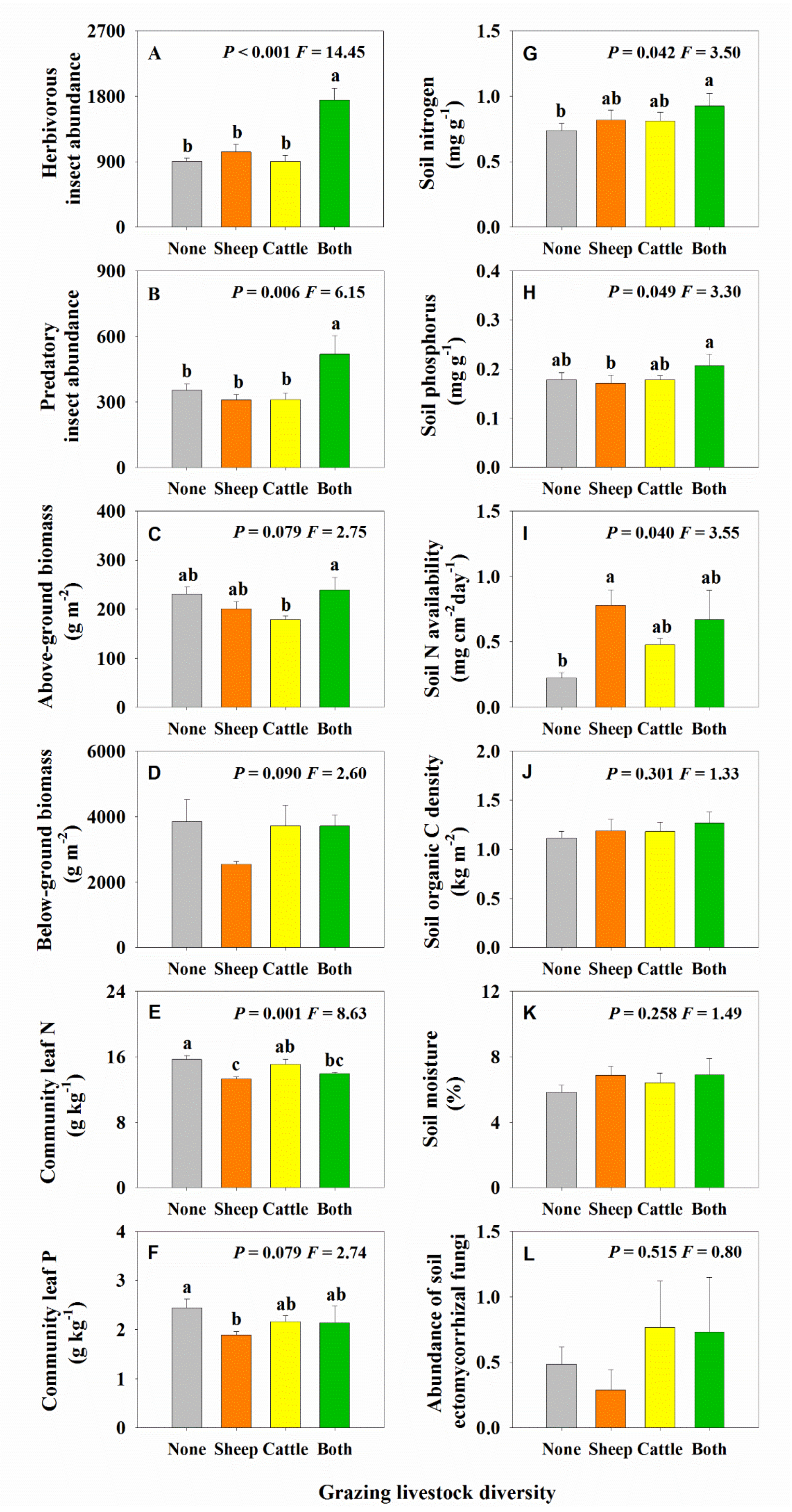
A
P < 0.001 F = 14.45
Herbivorous insect abundance
G
P = 0.042 F = 3.50
Soil nitrogen (mg g-1)
B
P = 0.006 F = 6.15
Predatory insect abundance
H
P = 0.049 F = 3.30
Soil phosphorus (mg g-1)
C
P = 0.079 F = 2.75
Above-ground biomass (g m-2)
I
P = 0.040 F = 3.55
Soil N availability (mg cm-2 day-1)
D
P = 0.090 F = 2.60
Below-ground biomass (g m-2)
J
P = 0.301 F = 1.33
Soil organic C density (kg m-2)
E
P = 0.001 F = 8.63
Community leaf N (g kg-1)
K
P = 0.258 F = 1.49
Soil moisture (%)
F
P = 0.079 F = 2.74
Community leaf P (g kg-1)
L
P = 0.515 F = 0.80
Abundance of soil ectomycorrhizal fungi
None Sheep Cattle Both
Grazing livestock diversity

Fig. S7. The effects of grazing livestock diversity on herbivorous insect abundance (*A*), predatory insect abundance (*B*), above-ground plant biomass (*C*), below-ground root biomass (*D*), plant community leaf N (*E*), plant community leaf P (*F*), soil total nitrogen (*G*), soil total phosphorus (*H*), soil N availability (*I*), soil organic C density (*J*), soil moisture (*K*), abundance of soil ectomycorrhizal fungi (*L*). Different lowercase letters within panels indicate significant ($P < 0.1$) differences between treatment means, after using Tukey's method to correct for multiple comparisons. Error bars represent ±1 SE.

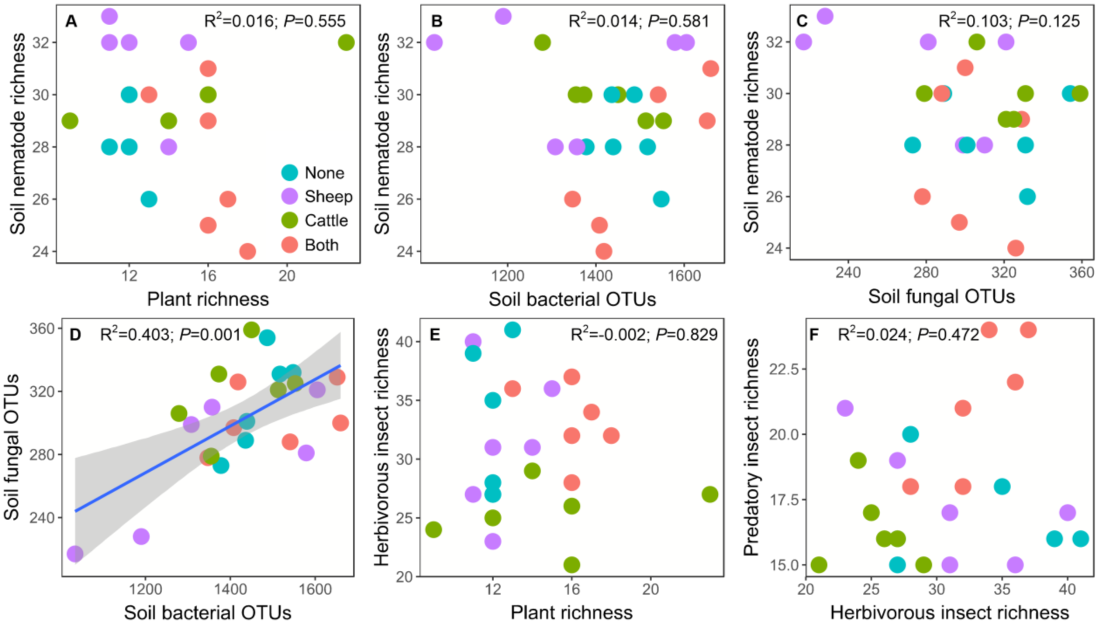

Fig. S8. The correlations between soil nematode richness and plant richness (*A*), soil bacterial OTUs (*B*), soil fungal OTUs (*C*), and relationship between soil bacterial OTUs and soil fungal OTUs (*D*), between plant richness and herbivorous insect richness (*E*), between herbivorous insect richness and predatory insect richness (*F*). The blue fitted lines are from OLS regression.

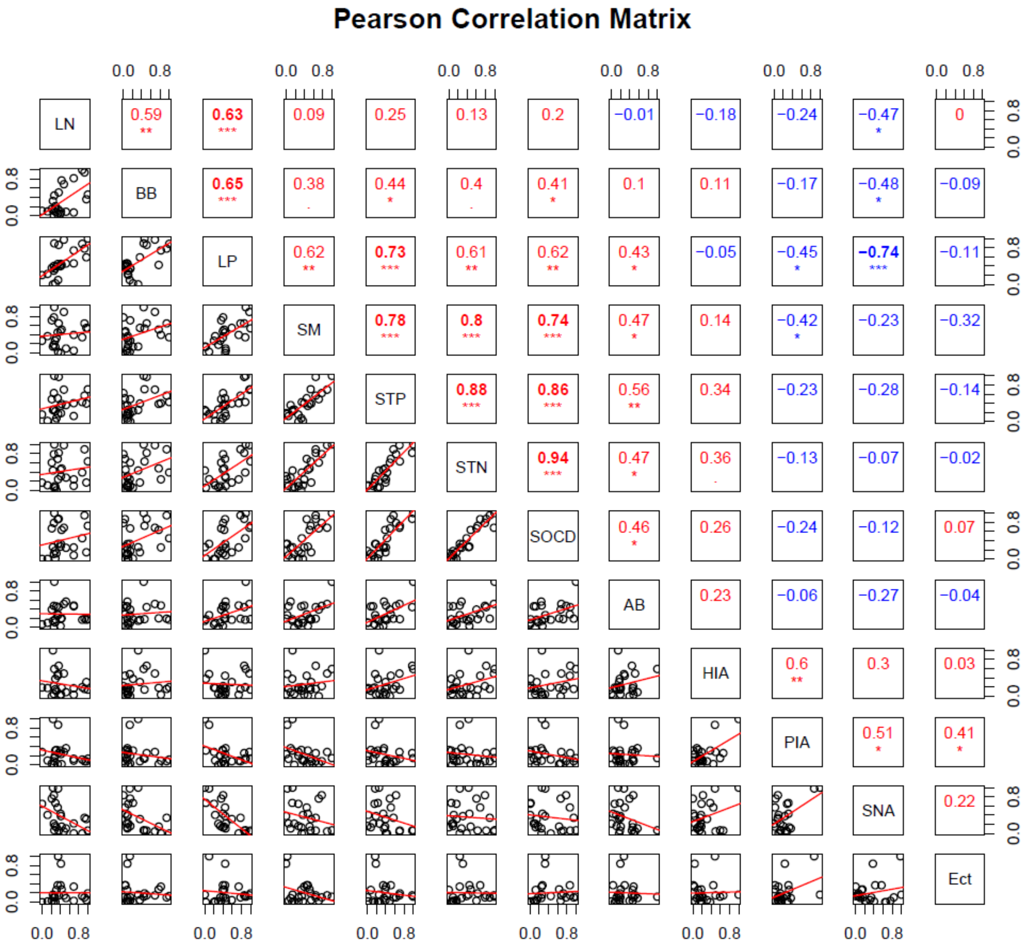

Fig. S9. Scatterplots matrices for the 12 ecosystem functions. LN = plant community leaf N; BB = below-ground root biomass; LP = plant community leaf P; SM = soil moisture; STP = soil total phosphorus; STN = soil total nitrogen; SOCD = soil organic C density; AB = above-ground plant biomass; HIA = herbivorous insect abundance; PIA = predatory insect abundance; SNA = soil N availability; Ect = abundance of soil ectomycorrhizal fungi. The upper triangular matrix shows the pairwise relationships among functions. *P*-values of the correlation coefficient are as follows: $^{***}P<0.001$, $^{**}P<0.01$, $^{*}P<0.05$.

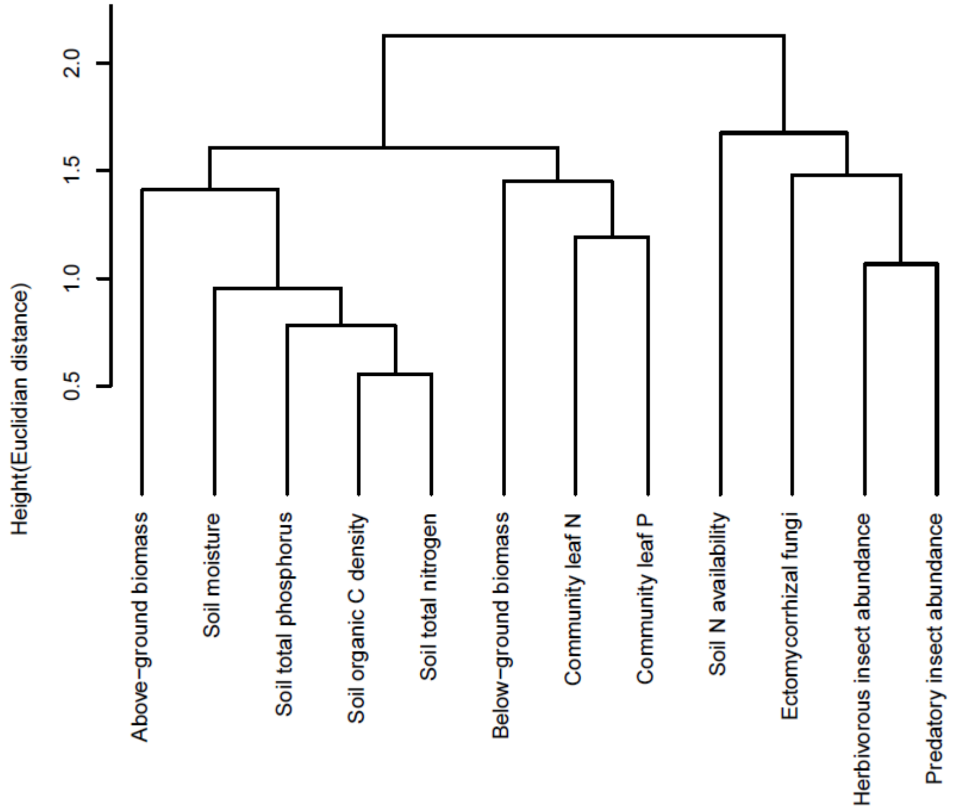

Fig. S10. The cluster analysis of 12 ecosystem functions.

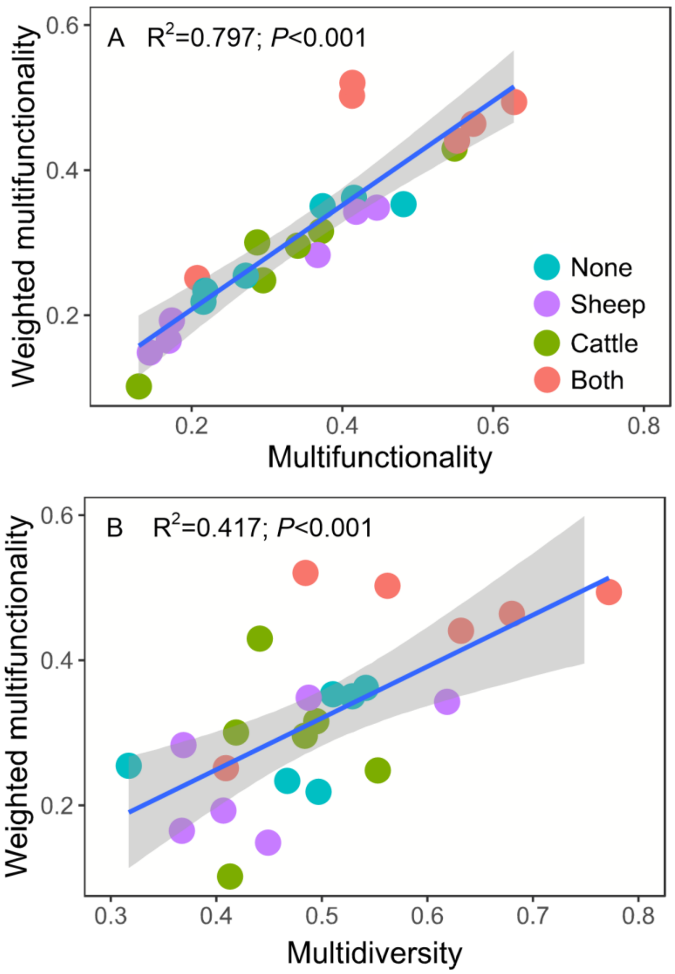

Fig. S11. The relationships between multifunctionality (EMF) and the weighted EMF (A), and between the weighted EMF and multidiversity (B). The weighted EMF is calculated as the average of all these functions after weighting each of soil C, N, P and moisture with a weight of 0.25 and then summing the four down-weighted functions into a single combined standardized variable, and weighting each of leaf N and P with a weight of 0.5 and then summing the two down-weighted functions into a single combined standardized variable, so that these correlated variables have a combined weight of only 1.

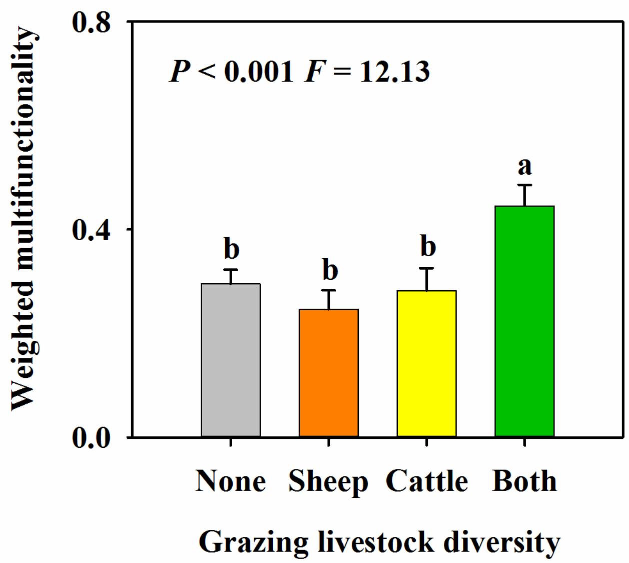

Fig. S12. The effects of grazing livestock diversity on the weighted ecosystem multifunctionality.

Table S1. The theoretical information on all 12 functions and their importance.

Ecosystem functions	Importance
**Above-ground functions**	
Aboveground herbivorous insect abundance	Biomass of high trophic organisms-herbivorous insects and predatory insects.
Aboveground predatory insect abundance	
Plant community leaf N	The key ecosystem processes that sustain human welfare, supply important nutrient sources for livestock herbivores (e.g., proteins and energy) (1), and plays major roles in the global carbon cycle (2-3).
Plant community leaf P	
Above-ground plant biomass	
**Below-ground functions**	
Below-ground root biomass	A key ecosystem process that supports belowground functionality
Soil total N	Build-up of nutrient pools that most frequently limit the primary production in grassland ecosystems.
Soil total P	
In situ soil N availability	Provide information on the production of available N within our plots.
Soil organic C density	Soil carbon-fixation function, climate regulation.
Soil moisture	Water regulation.
Abundance of soil ectomycorrizal fungi	Mycorrhizal colonization (4).

Table S2. Heatmap of correlation (Pearson) between all diversity and function combination. The numbers in the table are R value. The shading from white to blue represents gradation from low to high positive correlation. The shading from white to red represents gradation from low to high negative correlation. EMF includes all the 12 functions. Above-ground MF includes herbivorous insect and predatory insect abundance, above-ground biomass and plant community leaf N and P. Below-ground MF includes below-ground root biomass, soil N availability, soil total nitrogen, soil total phosphorus, soil organic C density, soil moisture and abundance of soil ectomycorrhizal fungi. Soil MF includes soil N availability, soil total nitrogen, soil total phosphorus, soil organic C density, soil moisture and soil ectomycorrhizal fungi. Plant MF includes plant above- and below-ground biomass, and plant community leaf N and P. Soil nutrients include soil N availability, soil total nitrogen, soil total phosphorus, soil organic C density and soil moisture. Plant nutrients include plant community leaf N and P. Plant biomass includes above-ground plant biomass and below-ground root biomass.

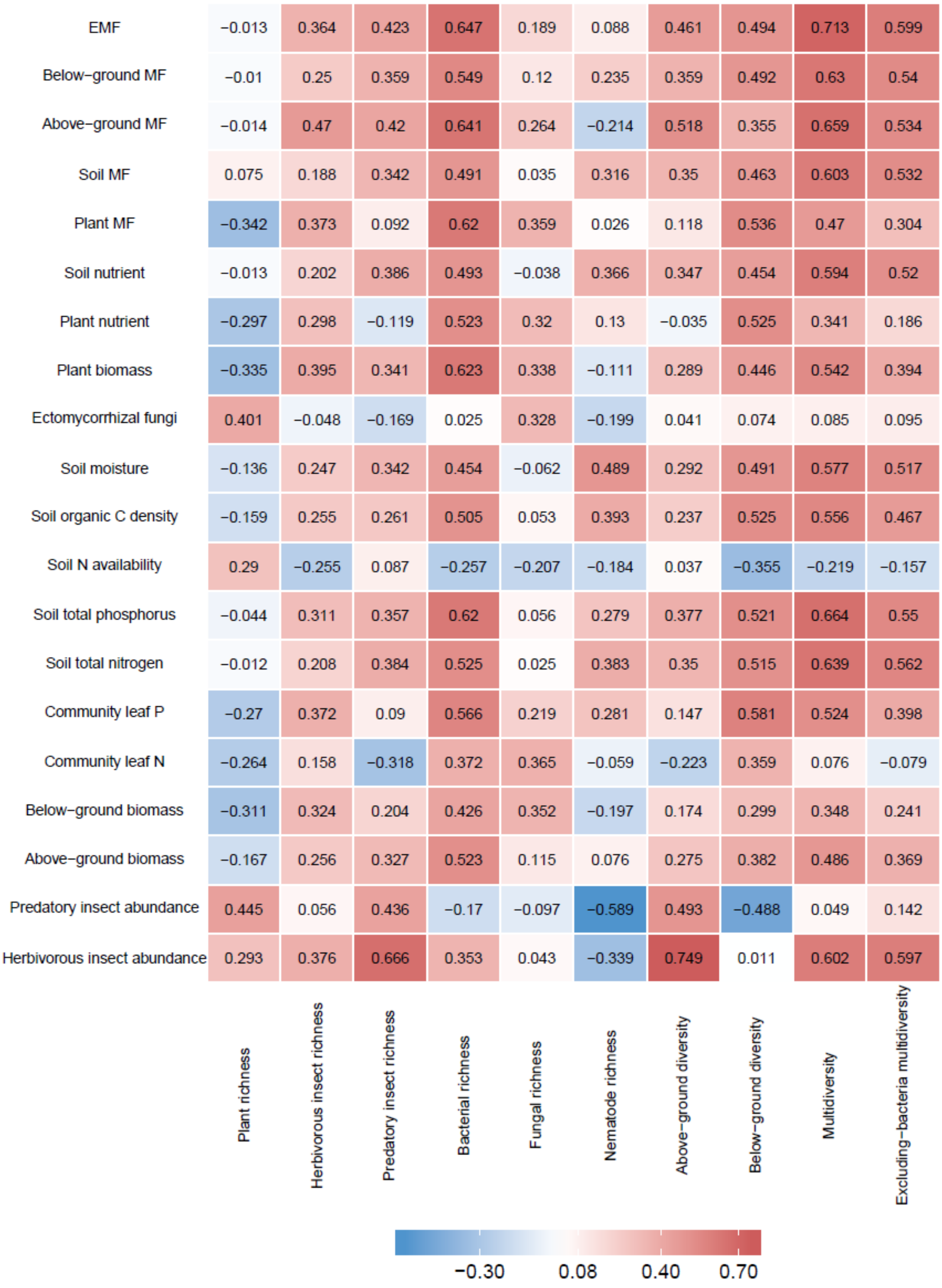
EMF −0.013 0.364 0.423 0.647 0.189 0.088 0.461 0.494 0.713 0.599
Below−ground MF −0.01 0.25 0.359 0.549 0.12 0.235 0.359 0.492 0.63 0.54
Above−ground MF −0.014 0.47 0.42 0.641 0.264 −0.214 0.518 0.355 0.659 0.534
Soil MF 0.075 0.188 0.342 0.491 0.035 0.316 0.35 0.463 0.603 0.532
Plant MF −0.342 0.373 0.092 0.62 0.359 0.026 0.118 0.536 0.47 0.304
Soil nutrient −0.013 0.202 0.386 0.493 −0.038 0.366 0.347 0.454 0.594 0.52
Plant nutrient −0.297 0.298 −0.119 0.523 0.32 0.13 −0.035 0.525 0.341 0.186
Plant biomass −0.335 0.395 0.341 0.623 0.338 −0.111 0.289 0.446 0.542 0.394
Ectomycorrhizal fungi 0.401 −0.048 −0.169 0.025 0.328 −0.199 0.041 0.074 0.085 0.095
Soil moisture −0.136 0.247 0.342 0.454 −0.062 0.489 0.292 0.491 0.577 0.517
Soil organic C density −0.159 0.255 0.261 0.505 0.053 0.393 0.237 0.525 0.556 0.467
Soil N availability 0.29 −0.255 0.087 −0.257 −0.207 −0.184 0.037 −0.355 −0.219 −0.157
Soil total phosphorus −0.044 0.311 0.357 0.62 0.056 0.279 0.377 0.521 0.664 0.55
Soil total nitrogen −0.012 0.208 0.384 0.525 0.025 0.383 0.35 0.515 0.639 0.562
Community leaf P −0.27 0.372 0.09 0.566 0.219 0.281 0.147 0.581 0.524 0.398
Community leaf N −0.264 0.158 −0.318 0.372 0.365 −0.059 −0.223 0.359 0.076 −0.079
Below−ground biomass −0.311 0.324 0.204 0.426 0.352 −0.197 0.174 0.299 0.348 0.241
Above−ground biomass −0.167 0.256 0.327 0.523 0.115 0.076 0.275 0.382 0.486 0.369
Predatory insect abundance 0.445 0.056 0.436 −0.17 −0.097 −0.589 0.493 −0.488 0.049 0.142
Herbivorous insect abundance 0.293 0.376 0.666 0.353 0.043 −0.339 0.749 0.011 0.602 0.597
Plant richness
Herbivorous insect richness
Predatory insect richness
Bacterial richness
Fungal richness
Nematode richness
Above−ground diversity
Below−ground diversity
Multidiversity
Excluding−bacteria multidiversity
−0.30 0.08 0.40 0.70

References

1. Binder S et al. (2018) Grassland biodiversity can pay. *Proc Natl Acad Sci USA* 115: 3876–3881.
2. Wardle D A et al. (2004) Ecological linkages between aboveground and belowground biota. *Science* 304:1629–1633.Hill AVS (1991) HLA associations with malaria in Africa: Some implications for MHC evolution. *Molecular Evolution of the Major Histocompatibility Complex*, eds Klein J, Klein D (Springer, Heidelberg), pp 403–420.
3. Pettorelli N et al. (2005) Using the satellite-derived NDVI to assess ecological responses to environmental change. *Trends Ecol Evol* 20: 503–510.
4. Soliveres S et al. (2016) Biodiversity at multiple trophic levels is needed for ecosystem multifunctionality. *Nature* 536:456–459.
